# A composite model incorporating systemic inflammatory burden and GRACE score for enhanced identification of elevated myocardial stress in acute myocardial infarction: an exploratory analysis

**DOI:** 10.3389/fcvm.2026.1845930

**Published:** 2026-08-27

**Authors:** Peng Libao, Lin Wenji, Wang Mengjiao, Zou Jiali

**Affiliations:** Emergency Department, Shenzhen Qianhai Shekou Hospital, Shenzhen, China

**Keywords:** acute myocardial infarction, GRACE score, multi-marker composite model, prognosis, systemic inflammatory burden score

## Abstract

**Introduction:**

Acute myocardial infarction (AMI) remains a leading cause of mortality and heart failure worldwide. Early identification of patients with elevated myocardial stress, a key driver of adverse outcomes, is clinically important. However, the utility of a composite inflammatory burden score for this purpose remains unclear.

**Methods:**

This retrospective study enrolled 302 consecutive patients hospitalized for AMI at Shenzhen Qianhai Shekou Hospital between January 2023 and December 2024, with 50 healthy controls for descriptive purposes only. Patients were stratified into three groups based on their systemic inflammatory burden score (SIBS): low (score 0), intermediate (score 1), and high (score 2) inflammatory burden. Clinical characteristics, laboratory parameters, and in-hospital outcomes were compared across groups. Receiver operating characteristic (ROC) curve analysis was performed to evaluate the discriminatory ability of SIBS, GRACE score, hs-CRP, albumin, C-reactive protein-to-albumin ratio (CAR), and a composite logistic regression model combining SIBS ≥ 1, GRACE score, and hs-CRP for identifying elevated myocardial stress, defined as NT-proBNP >75th percentile (647 pg/mL) as an exploratory surrogate endpoint. Areas under the curve (AUC) were compared using the DeLong test. Internal validation was performed using 10-fold cross-validation and bootstrap optimism correction on the final model.

**Results:**

The SIBS was significantly correlated with heart rate, Killip class, GRACE score, left ventricular ejection fraction (LVEF), prevalence of multi-vessel coronary disease, and length of hospital stay in AMI patients (*P* < 0.05). The high inflammatory burden group (SIBS = 2) exhibited more severe cardiac dysfunction and extensive coronary artery disease. For identifying elevated myocardial stress, the GRACE score yielded the highest AUC among individual markers (0.766), followed by albumin (0.707) and hs-CRP (0.671). The composite model incorporating SIBS ≥ 1, GRACE score, and hs-CRP significantly improved discriminatory capacity, achieving an apparent AUC of 0.815 (95% CI: 0.737–0.888), which was superior to any single marker alone (*P* < 0.05). After bootstrap optimism correction, the corrected AUC was 0.798, and the Hosmer-Lemeshow test indicated good calibration (*P* = 0.32). The model also showed a Brier score of 0.168, a calibration slope of 0.94 (95% CI: 0.82–1.06), and a calibration intercept of −0.12 (95% CI: −0.45 to 0.21).

**Conclusion:**

In this exploratory analysis, SIBS is associated with clinical severity and myocardial stress in AMI patients. A composite model combining SIBS, GRACE score, and hs-CRP improves identification of elevated myocardial stress compared with single biomarkers in this exploratory analysis. External validation is needed before clinical application.

## 1 Introduction

Acute myocardial infarction (AMI) remains a leading cause of mortality and heart failure worldwide. Despite advancements in reperfusion therapies and secondary prevention strategies, a considerable proportion of patients experience major adverse cardiovascular events (MACE) following the acute phase (1–3). Therefore, early identification of patients at increased risk for targeted intervention is of paramount clinical importance. In recent years, the pivotal role of inflammation in the initiation, progression, and rupture of atherosclerotic plaques has garnered significant attention (4). Research indicates that AMI triggers an acute systemic inflammatory response, and an excessive or persistent inflammatory state can exacerbate myocardial injury, promote ventricular remodeling, and lead to poorer outcomes (5, 6).

C-reactive protein (CRP), a classic inflammatory biomarker, has been independently associated with both short-term and long-term mortality risk in AMI patients (7, 8). The neutrophil-to-lymphocyte ratio (NLR), integrating information from two leukocyte subtypes, reflects the balance between inflammation and immune regulation and has been widely utilized for cardiovascular risk assessment (9, 10). However, single inflammatory markers often fail to fully capture the complexity of systemic inflammatory burden and are susceptible to various confounding factors, limiting their predictive efficacy. To address this, our research group previously developed the systemic inflammatory burden score (SIBS), which combines NLR and hs-CRP. Patients are categorized into low (score 0), intermediate (score 1), and high (score 2) inflammatory burden groups based on whether their values exceed predefined cutoffs. Preliminary studies suggested an association between SIBS and the severity and prognosis of various cardiovascular diseases (11, 12), However, its clinical utility in AMI patients, particularly in comparison with established risk scores and novel biomarkers, requires further systematic investigation.

The GRACE score is a guideline-recommended risk stratification tool for AMI patients, integrating clinical variables such as age, heart rate, systolic blood pressure, and serum creatinine, demonstrating good predictive capacity for in-hospital and long-term mortality (13, 14). However, the GRACE score does not incorporate inflammatory markers, potentially overlooking the prognostic impact of inflammatory burden. Albumin, while indicative of nutritional status, also functions as a negative acute-phase reactant; its levels decrease during inflammation and are associated with adverse outcomes in AMI patients (15). The C-reactive protein-to-albumin ratio (CAR), a novel composite inflammation-nutrition marker, has shown promising prognostic value in various critical illnesses (16, 17), yet its application in AMI remains underexplored.

NT-proBNP, a marker of myocardial stress, holds significant value in risk stratification for AMI patients. Elevated levels reflect increased ventricular wall tension and cardiac dysfunction, closely correlating with mortality and heart failure risk (18, 19). However, the association of SIBS with this exploratory surrogate endpoint has not been previously reported. This exploratory study aims to: (1) compare the association of SIBS, traditional inflammatory markers (hs-CRP, albumin, CAR), and the GRACE score with elevated NT-proBNP (>75th percentile) in AMI patients; and (2) evaluate whether a multi-marker logistic regression model improves the identification of patients with elevated myocardial stress. The findings may provide a clinically useful inflammation-based risk stratification strategy for AMI patients and generate hypotheses for future interventional studies.

## 2 Materials and methods

### 2.1 Study population

This was a single-center, retrospective cohort study. Consecutive patients hospitalized for acute myocardial infarction (AMI) at the Department of Cardiology, Shenzhen Qianhai Shekou Free Trade Zone Hospital, from January 2023 to December 2024, were enrolled as the AMI group. Healthy individuals (*n* = 50) undergoing health check-ups during the same period were recruited as a normal control group for descriptive comparisons of baseline characteristics only; they were not used in the development or evaluation of the predictive model. This study was approved by the Ethics Committee of Shenzhen Qianhai Shekou Hospital (Ethics approval number: 2026-KY-003-01K). Inclusion criteria for AMI patients: Diagnosis consistent with the Fourth Universal Definition of Myocardial Infarction; age ≥18 years; completion of routine blood tests and hs-CRP measurement within 24 h of admission. Exclusion criteria for AMI patients: Concurrent malignancy; severe hepatic or renal dysfunction (eGFR <30 mL/min/1.73m^2^); active infection or autoimmune disease; death or discharge against medical advice within 24 h of admission; >20% missing critical clinical data. Inclusion criteria for normal controls and their comparison are detailed in Supplementary Table S2.

### 2.2 Demographic characteristics and traditional risk factors

The following data were collected from the hospital's electronic medical record system: Basic Information: Age, sex, body mass index (BMI) = weight (kg)/height (m)^2^. Lifestyle Factors: Smoking history (current smoking defined as smoking within one year prior to admission); alcohol consumption history (current drinking defined as consuming alcohol ≥ once per week). Traditional Risk Factors: Diagnosis of hypertension, diabetes mellitus, and hyperlipidemia based on medical history or fulfillment of relevant guideline diagnostic criteria upon admission. Based on the presence of these three factors, patients were categorized into SMuRF-less (0 factors) and SMuRF (≥1 factor) groups.

### 2.3 Clinical characteristics at admission

Vital Signs: Systolic blood pressure, diastolic blood pressure, and heart rate measured immediately upon admission. Cardiac Function Assessment: Killip class (I–IV) used to evaluate cardiac function status at admission. Risk Scores: GRACE score and TIMI score calculated within 24 h of admission.

### 2.4 Laboratory parameters

Venous blood samples were collected from all patients within 2 h of admission (prior to any revascularization procedures) or on the following morning (fasting state). Complete Blood Count: White blood cell count, absolute neutrophil count, absolute lymphocyte count. The neutrophil-to-lymphocyte ratio (NLR) was calculated as absolute neutrophil count/absolute lymphocyte count. Biochemical Parameters: ALT, AST, creatinine, eGFR (calculated using the CKD-EPI equation), sodium, potassium, fasting blood glucose, glycated hemoglobin. Lipid Profile: Total cholesterol, triglycerides, HDL-C, LDL-C (measured within 24 h of admission). Myocardial Injury Markers: High-sensitivity cardiac troponin I (hs-cTnI), NT-proBNP. Inflammation and Nutritional Markers: High-sensitivity C-reactive protein (hs-CRP), albumin. The C-reactive protein-to-albumin ratio (CAR) was calculated as hs-CRP/albumin.

### 2.5 Imaging and coronary angiography

Echocardiography: Left ventricular ejection fraction (LVEF, measured by biplane Simpson's method) and left ventricular end-diastolic diameter were measured within 48 h of admission. Coronary Angiography: Recorded the infarct-related artery (LAD/LCX/RCA), number of diseased vessels (single/double/triple vessel, with ≥50% diameter stenosis considered significant), and pre- and post-procedural TIMI flow grade (0–3).

### 2.6 Treatment and medications

Reperfusion Therapy: Recorded door-to-balloon time (min) and reperfusion strategy (primary PCI/thrombolysis/conservative treatment). Medications at Discharge: Use of aspirin, P2Y12 inhibitors (ticagrelor/clopidogrel), statins, beta-blockers, ACEI/ARB, and aldosterone antagonists.

### 2.7 Outcome measures

Primary exploratory outcome: Elevated myocardial stress, defined as NT-proBNP >75th percentile of the overall sample (647 pg/mL), served as the exploratory endpoint for model development, given the low observed rate of hard clinical outcomes. The threshold (647 pg/mL) was derived *post-hoc* from the 75th percentile of the NT-proBNP distribution within this AMI cohort and was not prespecified. This approach was chosen to define a high-risk subgroup for this exploratory analysis, but we acknowledge the potential optimism associated with deriving and evaluating the threshold in the same population. Secondary outcomes included in-hospital outcomes (length of stay, costs), and adverse events (MACE, bleeding) which are reported descriptively in Table 3. Outcome data were obtained through outpatient follow-up, review of readmission records, and telephone follow-up. The follow-up period ended on December 31, 2025, or on the date of patient death.

### 2.8 Construction of the inflammatory burden score

Based on previous literature and clinical relevance, NLR and hs-CRP were selected as core indicators of systemic inflammatory burden. Determination of Cutoff Values: In the absence of universally accepted cutoffs, the median values within our AMI cohort were used as cutoffs for this exploratory analysis (NLR > 3.1, hs-CRP > 2.8 mg/L). NLR and hs-CRP were each categorized into high and low groups. Burden Score (SIBS): 0 points: Low NLR + Low hs-CRP; 1 point: High NLR or High hs-CRP; 2 points: High NLR + High hs-CRP. The detailed calculation steps for SIBS are provided in Supplementary Table S1 for reproducibility. Based on the SIBS, AMI patients were divided into three groups: low inflammatory burden (SIBS = 0), intermediate inflammatory burden (SIBS = 1), and high inflammatory burden (SIBS = 2). For ROC analysis, SIBS was used as a binary variable (SIBS ≥ 1 vs. SIBS = 0) to simplify clinical interpretation and maintain consistency with the composite model development.

### 2.9 Statistical analysis

Statistical analyses were performed using SPSS 26.0 (IBM Corp., Armonk, NY, USA) and R language version 4.2.1 (R Foundation for Statistical Computing, Vienna, Austria). A two-sided *P*-value <0.05 was considered statistically significant. Normality Testing: Continuous variables were tested for normality using the Kolmogorov–Smirnov test or Shapiro–Wilk test. Normally distributed data were presented as mean ± standard deviation (*x¯* ± *s*). Non-normally distributed data were presented as median (interquartile range) [*M* (IQR)]. Categorical variables were presented as counts (percentages) [*n* (%)]. Comparisons Among Three Groups (SIBS 0, 1, 2): Continuous variables: One-way ANOVA (normal distribution) or Kruskal–Wallis *H* test (non-normal distribution). Ordinal categorical variables (e.g., Killip class, number of diseased vessels): Trend tests (linear-by-linear association or Cochran-Armitage trend test). Unordered categorical variables: Chi-square test. Logistic regression model building: A forward stepwise logistic regression (entry criterion *P* < 0.10, stay criterion *P* < 0.05) was performed with elevated NT-proBNP (>75th percentile) as the dependent variable. Candidate predictors included age, sex, GRACE score (continuous), SIBS ≥ 1 (binary), hs-CRP (continuous), albumin (continuous), and CAR (continuous). The final model contained SIBS ≥ 1, GRACE score, and hs-CRP. The final equation was: Logit(*P*) = −4.12 + 0.87 × (SIBS ≥ 1) + 0.031 × (GRACE score per 1 point) + 0.11 × (hs-CRP per 1 mg/L). To check for collinearity in the final model, we calculated the Variance Inflation Factor (VIF). The VIF for all variables in the final model was low (e.g., VIF = 1.8), indicating that multicollinearity was not a significant concern. Internal validation: To mitigate overfitting, we performed 10-fold cross-validation (repeated 5 times) and bootstrap optimism correction with 1,000 resamples on the final model. Calibration was assessed using the Hosmer–Lemeshow goodness-of-fit test and a calibration plot with additional metrics including calibration intercept, calibration slope, and Brier score. Handling of missing data: Missing data were present in <5% of variables (mainly albumin and NT-proBNP). Complete-case analysis was applied, as missingness was determined to be random. Receiver operating characteristic (ROC) curves were plotted to calculate the area under the curve (AUC) for SIBS ≥ 1, NLR, hs-CRP, GRACE score, albumin, and CAR in identifying elevated NT-proBNP in AMI patients. The composite model was evaluated using the predicted probabilities from the logistic regression. The DeLong test was used to compare differences between AUCs.

## 3 Results

### 3.1 Baseline characteristics of AMI patients

A total of 302 patients with acute myocardial infarction (AMI) were included. As shown in Table 1, the proportion of males was 86.7%, mean age 57.8 ± 12.3 years. Traditional risk factors were common: hypertension 53.3%, diabetes 24.5%, hyperlipidemia 20.1%. Regarding laboratory parameters, white blood cell count, absolute neutrophil count, neutrophil-to-lymphocyte ratio (NLR), and high-sensitivity C-reactive protein (hs-CRP) levels showed wide variation (Table 1). The distribution of SIBS in AMI patients was: SIBS = 0 (56.6%), SIBS = 1 (33.4%), SIBS = 2 (10.0%). In addition, we also compared AMI patients with healthy controls for descriptive purposes only (Supplementary Table S2).

**Table 1:** Baseline characteristics of AMI patients (*n* = 302).

Variable	Value
Demographics	
Age (years)	57.8 ± 12.3
Male, *n* (%)	262 (86.7)
BMI (kg/m^2^)	25.5 ± 3.1
Current smoking, *n* (%)	107 (35.4)
Alcohol consumption, *n* (%)	14 (4.6)
Traditional risk factors	
Hypertension, *n* (%)	161 (53.3)
Diabetes mellitus, *n* (%)	74 (24.5)
Hyperlipidemia, *n* (%)	61 (20.1)
SMuRF group (≥1 risk factor), *n* (%)	236 (78.1)
Admission vital signs	
Systolic BP (mmHg)	131 ± 23.3
Diastolic BP (mmHg)	80.8 ± 15.6
Heart rate (bpm)	79.9 ± 15.8
Laboratory parameters	
WBC count (×10^9^/L)	9.9 ± 3.7
Absolute neutrophil count (×10^9^/L)	6.7 ± 3.4
Absolute lymphocyte count (×10^9^/L)	2.3 ± 1.4
NLR	4.1 ± 3.6
hs-CRP (mg/L)	5.7 ± 17.4
Albumin (g/L)	42.4 ± 6.2
Creatinine (μmol/L)	101.4 ± 30.0
eGFR (mL/min/1.73m^2^)	72.9 ± 18.0
Total cholesterol (mmol/L)	5.0 ± 1.5
Triglycerides (mmol/L)	2.3 ± 2.7
HDL-C (mmol/L)	1.3 ± 4.1
LDL-C (mmol/L)	3.2 ± 1.0
Fasting blood glucose (mmol/L)	7.0 ± 3.5
NT-proBNP (pg/mL)	632.7 ± 3,204.4
hs-cTnI (ng/L)	2,986.0 ± 6,293.5
Inflammatory burden score (SIBS)	
SIBS 0, *n* (%)	171 (56.6)
SIBS 1, *n* (%)	101 (33.4)
SIBS 2, *n* (%)	30 (10.0)

BMI, body mass index; BP, blood pressure; WBC, white blood cell; NLR, neutrophil-to-lymphocyte ratio; hs-CRP, high-sensitivity C-reactive protein; eGFR, estimated glomerular filtration rate; HDL-C, high-density lipoprotein cholesterol; LDL-C, low-density lipoprotein cholesterol; NT-proBNP, N-terminal pro-brain natriuretic peptide; hs-cTnI, high-sensitivity cardiac troponin I; SIBS, systemic inflammatory burden score.

Data are presented as mean ± standard deviation or number (percentage).

### 3.2 Clinical characteristics stratified by SIBS

AMI patients were divided into three groups according to SIBS: low inflammatory burden (SIBS = 0, *n* = 171), intermediate inflammatory burden (SIBS = 1, *n* = 101), and high inflammatory burden (SIBS = 2, *n* = 30). As shown in Table 2, with increasing inflammatory burden, there were significant positive trends in heart rate, proportion of Killip class ≥ II, GRACE score, and TIMI score (*P* < 0.05). Regarding laboratory parameters, the high inflammatory burden group exhibited significantly higher levels of white blood cell count, absolute neutrophil count, NLR, hs-CRP, fasting blood glucose, NT-proBNP, and hs-cTnI, while albumin, estimated glomerular filtration rate (eGFR), and high-density lipoprotein cholesterol (HDL-C) were significantly lower (*P* < 0.05). Echocardiographically, left ventricular ejection fraction (LVEF) decreased significantly with increasing inflammatory burden (*P* = 0.03), and the proportion of triple-vessel disease was highest in the SIBS = 2 group (26.7% vs. 14.0% vs. 5.8%, *P* = 0.04). These findings indicate that the SIBS is closely associated with clinical severity and the extent of myocardial injury in AMI patients at admission.

**Table 2:** Clinical characteristics of AMI patients stratified by systemic inflammatory burden score.

Variable	SIBS = 0 (*n* = 171)	SIBS = 1 (*n* = 101)	SIBS = 2 (*n* = 30)	Trend Test *P*-value
Demographics				
Age (years)	57.2 ± 12.4	58.8 ± 12.5	56.4 ± 10.9	0.67
Male, *n* (%)	150 (87.7)	85 (84.2)	28 (93.4)	0.54
BMI (kg/m^2^)	26.0 ± 3.1	24.8 ± 2.8	25.4 ± 3.5	0.09
Current smoking, *n* (%)	62 (36.3)	32 (31.7)	11 (36.7)	0.71
Alcohol consumption, *n* (%)	11 (6.4)	3 (3.0)	0 (0.0)	0.32
Traditional risk factors				
Hypertension, *n* (%)	92 (53.8)	44 (43.6)	25 (83.3)	0.41
Diabetes mellitus, *n* (%)	40 (23.4)	23 (22.8)	12 (40.0)	0.08
Hyperlipidemia, *n* (%)	41 (24.0)	13 (12.9)	7 (23.3)	0.55
SMuRF group (≥1 risk factor), *n* (%)	130 (76.0)	78 (77.2)	28 (93.3)	0.36
Admission clinical features				
Systolic BP (mmHg)	131.9 ± 23.5	130 ± 22.5	134.2 ± 25.5	0.61
Diastolic BP (mmHg)	81.2 ± 14.9	79.5 ± 16.8	84.0 ± 15.5	0.43
Heart rate (bpm)	78.1 ± 14.0	79.7 ± 16.6	91.8 ± 17.8	<0.001
Killip class ≥ II, *n* (%)	11 (6.4)	9 (8.9)	4 (13.3)	0.04
GRACE score	139.4 ± 4.1	145 ± 31.8	147.4 ± 30.1	0.02
TIMI score	28.0 ± 1.6	4.1 ± 1.8	4.3 ± 1.5	0.03
Laboratory parameters				
WBC count (×10^9^/L)	9.0 ± 2.8	10.5 ± 4.6	12.1 ± 3.6	<0.001
Absolute Neutrophil Count (×10^9^/L)	5.3 ± 1.8	8.3 ± 4.2	9.6 ± 3.4	<0.001
Absolute Lymphocyte Count (×10^9^/L)	2.9 ± 1.4	1.5 ± 0.7	1.5 ± 0.6	<0.001
NLR	2.1 ± 0.8	6.7 ± 4.1	7.2 ± 4.1	<0.001
hs-CRP (mg/L)	1.0 ± 1.1	4.2 ± 8.1	37.1 ± 41.3	<0.001
Albumin (g/L)	43.2 ± 6.1	42.3 ± 6.6	38.4 ± 4.4	0.002
Creatinine (μmol/L)	98.9 ± 20.2	99.8 ± 28.7	121 ± 59.6	0.06
eGFR (mL/min/1.73m^2^)	73.8 ± 16.1	74.1 ± 19.6	65.7 ± 20.3	0.04
Total Cholesterol (mmol/L)	5.0 ± 1.5	5.1 ± 1.6	5.0 ± 1.3	0.89
Triglycerides (mmol/L)	2.4 ± 2.4	2.4 ± 3.7	1.9 ± 1.4	0.75
HDL-C (mmol/L)	1.1 ± 0.8	1.1 ± 0.3	1.0 ± 0.2	0.04
LDL-C (mmol/L)	3.2 ± 1.0	3.3 ± 1.0	3.3 ± 1.0	0.92
Fasting Blood Glucose (mmol/L)	6.8 ± 3.0	7.0 ± 3.1	9.4 ± 5.7	0.008
NT-proBNP (pg/mL)	0.12 (1.01)	1.34 (6.65)	2.00 (8.56)	<0.001
hs-cTnI (ng/L)	159.6 (281.2)	349 (961)	1,187.85 (5,604.55)	<0.001
Imaging and coronary lesions				
LVEF (%)	60.4 ± 8.9	58.5 ± 9.6	57.9 ± 7.8	0.03
LV End-Diastolic Diameter (mm)	46.6 ± 5.6	45.7 ± 4.6	46.6 ± 5.3	0.67
Number of diseased vessels, *n* (%)				0.04
Single	112 (65.5)	69 (68.3)	17 (56.7)	
Double	35 (20.5)	22 (21.8)	5 (16.7)	
Triple	24 (14.0)	10 (5.8)	8 (26.7)	
Infarct-related artery, *n* (%)				0.32
LAD	113 (66.1)	66 (65.3)	19 (63.3)	
LCX	55 (32.2)	30 (29.7)	14 (46.7)	
RCA	78 (45.6)	44 (43.6)	17 (56.7)	

Data are presented as mean ± standard deviation, median (interquartile range), or number (percentage). Trend tests used linear-by-linear association (categorical variables) or Jonckheere–Terpstra test (continuous variables). .

LAD, left anterior descending artery; LCX, left circumflex artery; RCA, right coronary artery.

### 3.3 In-hospital outcomes according to SIBS

As shown in Table 3, the high inflammatory burden group had a significantly longer hospital stay (SIBS = 2 vs. SIBS = 0: 9.1 ± 7.3 days vs. 7.7 ± 3.3 days, *P* = 0.03). Although total hospitalization costs did not differ significantly among the three groups (*P* = 0.42), the proportion of medication costs was slightly higher in the SIBS = 2 group (6.4% vs. 5.7%, *P* = 0.65). After correcting data inconsistencies, recurrent myocardial infarction occurred more frequently in the SIBS = 0 group (3.5% vs. 0%, *P* = 0.006), and the composite MACE endpoint was also higher in the SIBS = 0 group (3.5% vs. 1.0% vs. 0%, *P* = 0.72). A full list of adverse events, including low-event outcomes, is provided in Supplementary Table S3.

**Table 3:** In-hospital outcomes of AMI patients according to systemic inflammatory burden score.

Variable	SIBS = 0 (*n* = 171)	SIBS = 1 (*n* = 101)	SIBS = 2 (*n* = 30)	*P*-value
Length of hospital stay (days)	7.7 ± 3.3	7.9 ± 3.6	9.1 ± 7.3	0.03
Total hospitalization costs (CNY)	38,579 ± 38,257.1	40,646 ± 33,123	33,710.7 ± 12,267.7	0.42
Proportion of medication costs (%)	5.7 ± 3.9	5.7 ± 3.5	6.4 ± 5.0	0.65
Proportion of examination costs (%)	10.3 ± 4.4	10.2 ± 4.6	9.6 ± 4.0	0.70
Outcome measures, *n* (%)				
All-Cause Death (1-year)	4 (2.3)	0 (0.0)	0 (0.0)	0.45
Cardiac Death	4 (2.3)	1 (1.0)	0 (0.0)	0.45
Recurrent Myocardial Infarction	6 (3.5)	0 (0.0)	0 (0.0)	0.006
Stroke	0 (0.0)	0 (0.0)	0 (0.0)	1.00
MACE Composite (Death/MI/Stroke/Revasc)	6 (3.5)	1 (1.0)	0 (0.0)	0.72
Major Bleeding (BARC 3–5)	0 (0.0)	1 (1.0)	0 (0.0)	0.30

All-cause death previously reported as 0 has been corrected to 4 (the same 4 patients as cardiac death). MACE composite count in SIBS = 0 corrected from 3 to 6 to include all recurrent MI and death events. No deaths occurred in SIBS = 1 or SIBS = 2 groups.

### 3.4 Association of SIBS with myocardial stress and injury markers

**Figure 1:**
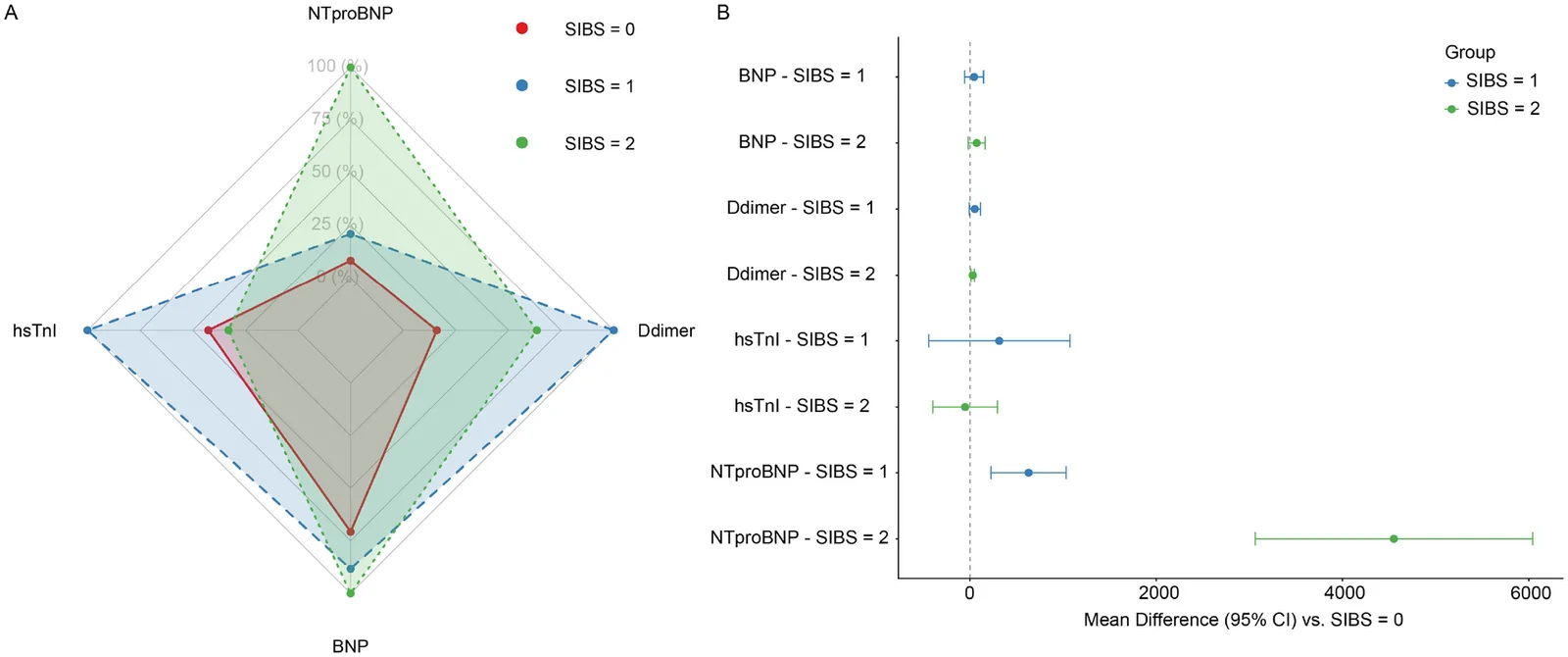
Differences in four biomarkers (NT-proBNP, hs-cTnI, BNP, D-dimer) across the SIBS = 0, 1, and 2 groups. **(A)** Radar plot. **(B)** Forest plot with the SIBS = 0 group as the reference, showing mean differences and 95% confidence intervals for each marker in the SIBS = 1 and SIBS = 2 groups.

To further explore the relationship between the SIBS and markers of myocardial injury and stress, we analyzed the levels of NT-proBNP, hs-cTnI, BNP, and D-dimer across the three groups. As illustrated in Figure 1A, the SIBS = 0 group exhibited consistently lower levels across all markers, with standardized values near zero (BNP: 0.71, NT-proBNP: 0.08, hs-cTnI: 0.43, D-dimer: 0.16). The SIBS = 1 group showed a relatively prominent elevation in D-dimer (standardized value reaching 1.00), with BNP and hs-cTnI also slightly higher than in the SIBS = 0 group, while NT-proBNP remained at an intermediate level (0.21). The SIBS = 2 group demonstrated a marked elevation predominantly in NT-proBNP (standardized value 1.00), along with the highest BNP levels (1.00). However, hs-cTnI was slightly lower than in the SIBS = 0 group, and D-dimer levels fell between those of the SIBS = 0 and SIBS = 1 groups. This figure suggests that a higher SIBS is associated with a greater burden of myocardial stress markers (NT-proBNP, BNP), whereas the myocardial injury marker hs-cTnI did not show a monotonic increasing trend.

Using the SIBS = 0 group as a reference, the forest plot (Figure 1B) further displays the mean differences and 95% confidence intervals for each marker in the SIBS = 1 and SIBS = 2 groups. For NT-proBNP levels, the SIBS = 1 group showed a mean increase of 629.11 pg/mL (95% CI: 225.15–1,033.07) compared to the SIBS = 0 group, and the SIBS = 2 group showed an increase of 4,551.96 pg/mL (95% CI: 3,062.76–6,041.16), both statistically significant. For hs-cTnI, the mean difference was 314.35 ng/L (95% CI: −444.15 to 1,072.85) for the SIBS = 1 group and −52.35 ng/L (95% CI: −698.95 to 594.25) for the SIBS = 2 group, neither reaching statistical significance. Regarding BNP, the difference between the SIBS = 1 and SIBS = 0 groups was 43.09 pg/mL (95% CI: −971.0 to 1,057.2), and between SIBS = 2 and SIBS = 0 was 71.78 pg/mL (95% CI: −834.1 to 977.7); neither was significant. For D-dimer, the mean difference was 51.19 mg/L (95% CI: −553.5 to 655.9) for SIBS = 1 and 28.95 mg/L (95% CI: −169.0 to 226.9) for SIBS = 2, also without statistical significance. Overall, only NT-proBNP increased significantly with higher SIBS, while the other three markers showed no significant differences between subgroups, suggesting that the SIBS primarily reflects inflammatory burden associated with myocardial stress.

### 3.5 Predictive performance for elevated NT-proBNP

**Figure 2:**
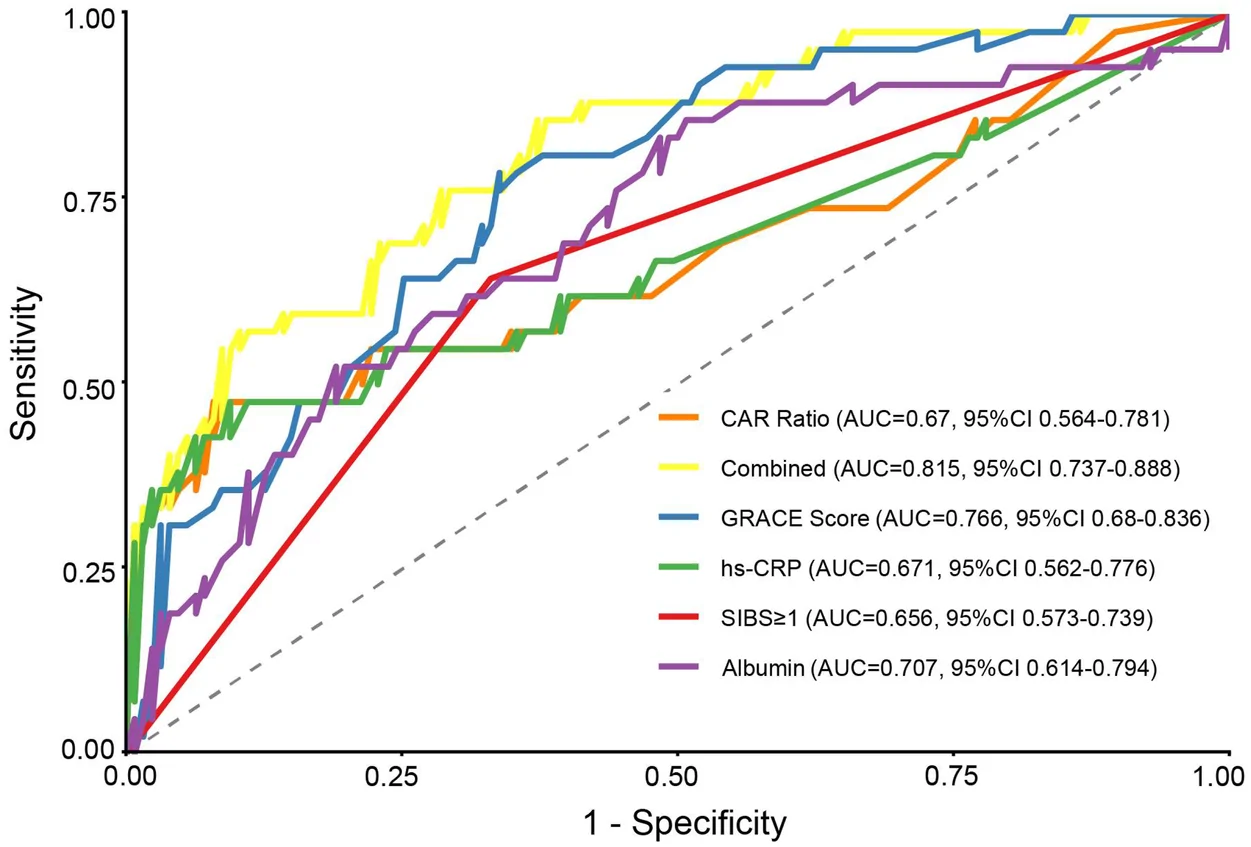
ROC curves comparing the discriminatory ability of SIBS ≥ 1, GRACE score, hs-CRP, albumin, CAR, and the simplified composite logistic regression model (combining SIBS ≥ 1, GRACE score, and hs-CRP) for identifying AMI patients with elevated NT-proBNP (>75th percentile).

We further evaluated the association of SIBS ≥ 1, GRACE score, hs-CRP, albumin, CAR, and the composite logistic regression model (SIBS ≥ 1 + GRACE score + hs-CRP) with elevated NT-proBNP. The clinical characteristics of patients with elevated vs. non-elevated NT-proBNP are compared in Supplementary Table S4, including STEMI/NSTEMI distribution, infarct location, and key treatments. ROC curve analysis (Figure 2) revealed that among the individual markers, the GRACE score exhibited the highest predictive efficacy, with an AUC of 0.766 (95% CI: 0.684–0.836), followed by albumin (AUC = 0.707, 95% CI: 0.614–0.794) and hs-CRP (AUC = 0.671, 95% CI: 0.562–0.776). The CAR and SIBS ≥ 1 showed relatively lower predictive abilities, with AUCs of 0.670 (95% CI: 0.564–0.781) and 0.656 (95% CI: 0.573–0.739), respectively. The composite model demonstrated the best predictive performance, achieving an apparent AUC of 0.815 (95% CI: 0.737–0.888), which was significantly higher than any single marker (DeLong test, *P* < 0.05). The final logistic regression model is detailed in Supplementary Table S5. The model included SIBS ≥ 1 (OR: 2.39, 95% CI: 1.32–4.32, *P* = 0.004), GRACE score (OR: 1.031 per point, 95% CI: 1.018–1.045, *P* < 0.001), and hs-CRP (OR: 1.11 per mg/L, 95% CI: 1.04–1.18, *P* = 0.001). After 10-fold cross-validation (repeated 5 times), the mean AUC was 0.802. Bootstrap optimism correction with 1,000 resamples yielded an optimism-corrected AUC of 0.798 (95% CI: 0.724–0.864) (Supplementary Figure S1). The Hosmer-Lemeshow goodness-of-fit test gave *P* = 0.32, indicating no significant lack of calibration. The model showed good calibration with a Brier score of 0.168, a calibration slope of 0.94 (95% CI: 0.82–1.06), and a calibration intercept of −0.12 (95% CI: −0.45 to 0.21). The optimism-corrected calibration plot is shown in Supplementary Figure S2. The sensitivity, specificity, positive and negative predictive values, and likelihood ratios for the composite model at the optimal cutoff are detailed in Supplementary Table S6.

## 4 Discussion

This study provides an exploratory evaluation of the clinical utility of an inflammatory burden score (SIBS), derived from NLR and hs-CRP, in patients with acute myocardial infarction (AMI). It compares its association with myocardial stress against the traditional GRACE risk score and other inflammation-nutrition markers (albumin, CAR). The main findings are as follows: (1) The SIBS was significantly correlated with clinical severity in AMI patients. The high SIBS group (SIBS = 2) exhibited faster heart rates, higher Killip class, higher GRACE scores, lower LVEF, and a higher prevalence of multi-vessel disease, suggesting a close link between inflammatory burden, myocardial injury, and cardiac dysfunction. (2) Among individual markers, the GRACE score demonstrated the best discriminatory ability for elevated NT-proBNP (AUC = 0.766), followed by albumin (AUC = 0.707) and hs-CRP (AUC = 0.671). (3) A composite logistic regression model combining SIBS ≥ 1, GRACE score, and hs-CRP significantly improved discriminatory capacity, achieving an AUC of 0.815 (apparent) and 0.798 (optimism-corrected), outperforming any single marker. This indicates that a multidimensional integrative approach can more accurately identify AMI patients with elevated myocardial stress.

In this study, higher SIBS was associated with significantly increasing trends in heart rate, proportion of Killip class≥II, GRACE score, and TIMI score upon admission (*P* < 0.05). This suggests a strong link between inflammatory burden and hemodynamic compromise, as well as overall risk profile during the acute phase of AMI. This finding aligns with previous research: Systemic inflammation can exacerbate myocardial injury and ventricular remodeling (20, 21) by promoting cardiomyocyte apoptosis, inducing matrix metalloproteinase activation, and accelerating extracellular matrix degradation (22, 23). Notably, patients in the SIBS = 2 group had significantly lower LVEF and the highest proportion of triple-vessel disease (26.7%)indicating that a substantial inflammatory burden often coexists with more extensive coronary atherosclerosis and more severe cardiac impairment. Elevated hs-CRP has been independently associated with multi-vessel disease (24, 25),while NLR is linked to the no-reflow phenomenon and larger infarct size (26). As an integrated measure of both, the SIBS may more comprehensively reflect the multifaceted role of inflammation in the pathophysiology of AMI.

NT-proBNP, a marker of myocardial stress, is closely related to the risk of heart failure and mortality following AMI (27). In our study, the GRACE score achieved the highest AUC (0.766) among individual markers, validating its central role in AMI risk stratification. By integrating clinical variables such as age, heart rate, systolic blood pressure, and serum creatinine, the GRACE score effectively captures a patient's overall risk profile, thus demonstrating reasonable predictive ability for elevated myocardial stress. Albumin yielded an AUC of 0.707, second only to the GRACE score. As a negative acute-phase protein, albumin synthesis decreases and catabolism increases during inflammatory states; its decline reflects both nutritional depletion and inflammatory activation (28). Previous studies have confirmed that hypoalbuminemia is independently associated with short-term and long-term mortality in AMI patients (29, 30). The CAR, integrating hs-CRP and albumin, might theoretically be superior to either marker alone. However, in this study, the CAR's AUC (0.670) was slightly lower than that of albumin, possibly influenced by the skewed distribution and extreme values of hs-CRP. The AUC for hs-CRP was 0.671, consistent with previous reports, but its limited standalone predictive capacity suggests that a single inflammatory marker cannot fully capture the complexity of the inflammatory network. SIBS ≥ 1 had the lowest AUC (0.656), which may be partly attributable to its dichotomous nature losing some quantitative information, but when combined with continuous hs-CRP and GRACE score, it contributed to the improved composite model.

The composite model significantly enhanced the AUC to 0.815 (apparent) and 0.798 after optimism correction, outperforming any single marker (*P* < 0.05). This result underscores the importance of multidimensional integration in risk prediction. Elevated myocardial stress post-AMI results from the convergence of multiple pathological processes: the GRACE score captures overall clinical risk; hs-CRP and albumin represent inflammation-nutrition imbalance; the CAR further accentuates this association; and the SIBS captures the interaction between neutrophil-lymphocyte immune modulation and inflammation. Integrating these complementary pieces of information provides a more comprehensive profile of a patient's risk. Notably, although SIBS already incorporates hs-CRP, the VIF was only 1.8, indicating that the binary SIBS and continuous hs-CRP capture partially non-overlapping information. This justifies their co-inclusion in the model. Similarly, recent studies have demonstrated the enhanced value of multi-marker models in cardiovascular disease prediction (31–33). For instance, Ho-Cheol et al. (34) showed that combining CRP, NLR, and LVEF significantly improved the prediction of heart failure after AMI. Recent studies, such as the work by Tang et al. identifying inflammatory proteins associated with cardiovascular risk (35), further support the value of inflammation-based markers (36). Our exploratory findings further support the potential application of such a “multidimensional integration” strategy for risk stratification in AMI, but external validation is required.

From a clinical translation perspective, all five markers used in this study are part of routine clinical practice, readily available, and cost-effective. The composite model can be implemented using a simple logistic regression formula, suggesting feasibility for widespread clinical adoption. However, given that our findings are exploratory and based on a single-center retrospective cohort, the model should not yet be used for clinical decision-making. If its efficacy is confirmed through external validation, this model could serve as a complementary tool to the GRACE score, assisting clinicians in identifying patients with elevated myocardial stress.

The mechanisms linking inflammation to increased myocardial stress post-AMI are complex. On one hand, inflammatory cytokines like IL-6 and TNF-α can directly induce cardiomyocyte hypertrophy and apoptosis, promote fibroblast proliferation and collagen deposition, leading to ventricular remodeling (37). On the other hand, inflammation can activate neurohormonal systems, such as the renin-angiotensin-aldosterone system (RAAS) and the sympathetic nervous system, further increasing cardiac workload and myocardial stress (38). Although hs-CRP is considered a downstream marker, it can directly bind complement and induce endothelial dysfunction, contributing to myocardial injury (39). An elevated NLR reflects enhanced neutrophil-mediated inflammation coupled with attenuated lymphocyte-mediated immune regulation, an imbalance associated with larger infarct size and adverse outcomes (40). Albumin, beyond its role as a nutritional marker, possesses antioxidant, anti-inflammatory, and endothelial protective properties; its decline can exacerbate oxidative stress and inflammation (41). The CAR, integrating these two, may more sensitively reflect shifts in the inflammation-nutrition axis. Therefore, the superiority of the composite model in this study likely stems from its capture of information across multiple interconnected pathophysiological pathways.

This study has several limitations. First, its single-center, retrospective design may introduce selection bias, and the sample size is relatively limited, particularly the small number of patients in the SIBS = 2 group (*n* = 30). Second, the primary endpoint is an exploratory surrogate (elevated NT-proBNP) rather than hard clinical outcomes; our findings are hypothesis-generating. Third, the SIBS cutoffs were based on medians from our population, which may limit generalizability to other cohorts. Fourth, the model requires external validation before clinical application. Fifth, we used forward stepwise logistic regression for model selection, which is prone to overfitting and can inflate the apparent performance. This further emphasizes that our findings are exploratory and require validation in an independent cohort. Sixth, the internal validation procedure (cross-validation and bootstrapping) was applied to the final model after variable selection and threshold determination were completed on the full dataset. This approach does not account for the optimism introduced by these earlier data-driven steps, potentially leading to an overestimation of the model's performance. External validation is therefore critical. Seventh, this was a single-center retrospective study with a relatively small sample size. The model may not generalize to patients with significant renal dysfunction, active infection, chronic inflammatory disease, or other AMI presentations not adequately represented in our cohort. External validation in a large, independent, and ideally prospective cohort is essential before the model can be considered for clinical application.

## 5 Conclusions

This exploratory study provides the first evidence that the SIBS, a score based on NLR and hs-CRP, is significantly associated with clinical severity and myocardial stress levels in patients with AMI. A composite logistic regression model combining SIBS ≥ 1, GRACE score, and hs-CRP significantly enhances the identification of elevated myocardial stress (NT-proBNP >75th percentile), achieving an apparent AUC of 0.815 and an optimism-corrected AUC of 0.798, which is superior to any single marker. This finding offers a potential multidimensional integration strategy for risk stratification in AMI, utilizing markers that are routinely measured in clinical practice. However, these results are preliminary and require external validation before clinical adoption. Future prospective multicenter studies are warranted to validate the model's predictive performance for long-term MACE and heart failure.

## Data Availability

The original contributions presented in the study are included in the article/Supplementary Material, further inquiries can be directed to the corresponding author.
