# Supplementary material for: A composite model incorporating systemic inflammatory burden and GRACE score for enhanced identification of elevated myocardial stress in acute myocardial infarction: an exploratory analysis
Source: Front Cardiovasc Med. 2026 Aug 27;13:1845930. doi: 10.3389/fcvm.2026.1845930 (PMC13558105; doi:10.3389/fcvm.2026.1845930)
Supplement: Supplementary file 1 [file Datasheet1.doc]

## Supplementary Table S1. Calculation of the systemic inflammatory burden score (SIBS)

| Step | Parameter | Cutoff Value | Points Assigned |
| --- | --- | --- | --- |
| 1 | Neutrophil-to-lymphocyte ratio (NLR) | ≤ 3.1 | 0 |
|  |  | > 3.1 | 1 |
| 2 | High-sensitivity C-reactive protein (hs-CRP) | ≤ 2.8 mg/L | 0 |
|  |  | > 2.8 mg/L | 1 |
| 3 | SIBS | Sum of points from Step 1 and Step 2 |  |

Notes: SIBS = 0: Low inflammatory burden; SIBS = 1: Intermediate inflammatory burden; SIBS = 2: High inflammatory burden. Cutoff values were derived from the median values of NLR and hs-CRP within the AMI cohort (NLR > 3.1, hs-CRP > 2.8 mg/L) for the purpose of this exploratory analysis. For binary analysis in ROC curves and logistic regression, SIBS was dichotomized as SIBS ≥ 1 (elevated inflammatory burden) versus SIBS = 0 (low inflammatory burden).

Supplementary Table S2. Baseline characteristics of AMI patients and healthy adults

| **Variable** | **Healthy**  **(n = 50)** | **AMI Patients**  **(n = 302)** | **Statistic** | ***P*-value** |
| --- | --- | --- | --- | --- |
| **Demographics** |  |  |  |  |
| Age (years) | 56.3 ± 12.6 | 57.8 ± 12.3 | t | 0.434 |
| Male, n (%) | 18 (36) | 262 (86.7) | χ² | **<0.001** |
| BMI (kg/m²) | 24.3 ± 3.4 | 25.5 ± 3.1 | t | **0.013** |
| Current smoking, n (%) | 11 (22) | 107 (35.4) | χ² | 0.145 |
| Alcohol consumption, n (%) | 2 (4) | 14 (4.6) | χ² | 0.79 |
| **Traditional risk factors** |  |  |  |  |
| Hypertension, n (%) | 16 (32) | 161 (53.3) | χ² | **0.0074** |
| Diabetes mellitus, n (%) | 6 (12) | 74 (24.5) | χ² | 0.0722 |
| Hyperlipidemia, n (%) | 1 (2) | 61 (20.1) | χ² | **<0.001** |
| SMuRF group (≥1 risk factor), n (%) | 26 (52) | 236 (78.1) | χ² | **<0.001** |
| **Admission vital signs** |  |  |  |  |
| Systolic BP (mmHg) | 128.9 ± 17.9 | 131 ± 23.3 | t | 0.489 |
| Diastolic BP (mmHg) | 76.0 ± 12.4 | 80.8 ± 15.6 | t | 0.036 |
| Heart rate (bpm) | 81.7 ± 12.9 | 79.9 ± 15.8 | t | 0.418 |
| **Laboratory parameters** |  |  |  |  |
| WBC count (×10⁹/L) | 5.9 ± 1.4 | 9.9 ± 3.7 | t | **<0.001** |
| Absolute neutrophil count (×10⁹/L) | 3.3 ± 1.0 | 6.7 ± 3.4 | t | **<0.001** |
| Absolute lymphocyte count (×10⁹/L) | 2.0 ± 0.7 | 2.3 ± 1.4 | t | 0.139 |
| NLR | 1.8 ± 0.8 | 4.1 ± 3.6 | MW | **<0.001** |
| hs-CRP (mg/L) | 1.4 ± 1.4 | 5.7 ± 17.4 | MW | **<0.001** |
| Albumin (g/L) | 44.5 ± 6.8 | 42.4 ± 6.2 | t | **0.03** |
| Creatinine (μmol/L) | 83.8 ± 132.0 | 101.4 ± 30.0 | t | 0.10 |
| eGFR (ml/min/1.73m²) | 76.5 ± 12.8 | 72.9 ± 18.0 | t | 0.18 |
| Total cholesterol (mmol/L) | 5.5 ± 1.3 | 5.0 ± 1.5 | t | 0.08 |
| Triglycerides (mmol/L) | 1.4 ± 0.7 | 2.3 ± 2.7 | t | 0.02 |
| HDL-C (mmol/L) | 1.4 ± 0.3 | 1.3 ± 4.1 | t | 0.12 |
| LDL-C (mmol/L) | 3.5 ± 1.0 | 3.2 ± 1.0 | t | 0.06 |
| Fasting blood glucose (mmol/L) | 5.4 ± 1.0 | 7.0 ± 3.5 | t | **<0.001** |
| NT-proBNP (pg/mL) | — | 632.7 ± 3204.4 | — | — |
| hs-cTnI (ng/L) | — | 2986.0 ± 6293.5 | — | — |
| **Inflammatory burden score (SIBS)** |  |  |  |  |
| SIBS 0, n (%) | 46 (92) | 171 (56.6) | χ² | **<0.001** |
| SIBS 1, n (%) | 4 (8) | 101 (33.4) |  |  |
| SIBS 2, n (%) | 0 (0) | 30 (10.0) |  |  |

Supplementary Table S3. Full list of adverse events

| Adverse event | SIBS = 0 (n=171) | SIBS = 1 (n=101) | SIBS = 2 (n=30) | Total (N=302) |
| --- | --- | --- | --- | --- |
| **All‑cause death (1‑year)** | 4 (2.3%) | 0 (0.0%) | 0 (0.0%) | 4 (1.3%) |
| Cardiac death | 4 (2.3%) | 1 (1.0%) | 0 (0.0%) | 5 (1.7%) |
| Recurrent myocardial infarction | 6 (3.5%) | 0 (0.0%) | 0 (0.0%) | 6 (2.0%) |
| Stroke (any) | 0 (0.0%) | 0 (0.0%) | 0 (0.0%) | 0 (0.0%) |
| Revascularization (PCI/CABG) | 0 (0.0%) | 0 (0.0%) | 0 (0.0%) | 0 (0.0%) |
| **MACE composite** (death/MI/stroke/revasc) | 6 (3.5%) | 1 (1.0%) | 0 (0.0%) | 7 (2.3%) |
| Major bleeding (BARC 3‑5) | 0 (0.0%) | 1 (1.0%) | 0 (0.0%) | 1 (0.3%) |
| **Heart failure hospitalization** | Not collected | Not collected | Not collected | Not collected |
| **Cardiogenic shock** | Not collected | Not collected | Not collected | Not collected |

## Supplementary Table S4. Clinical characteristics of AMI patients stratified by NT-proBNP level

| Variable | NT-proBNP ≤ 647 pg/mL (Non-elevated) (n = 227) | NT-proBNP > 647 pg/mL (Elevated) (n = 75) | *P*-value |
| --- | --- | --- | --- |
| Demographics |  |  |  |
| Age (years) | 56.8 ± 12.1 | 61.2 ± 11.8 | 0.02 |
| Male, n (%) | 198 (87.2) | 64 (85.3) | 0.68 |
| BMI (kg/m²) | 25.7 ± 3.0 | 24.9 ± 3.2 | 0.09 |
| Traditional risk factors |  |  |  |
| Hypertension, n (%) | 115 (50.7) | 46 (61.3) | 0.11 |
| Diabetes mellitus, n (%) | 51 (22.5) | 23 (30.7) | 0.15 |
| Hyperlipidemia, n (%) | 47 (20.7) | 14 (18.7) | 0.70 |
| SMuRF (≥1 risk factor), n (%) | 173 (76.2) | 63 (84.0) | 0.16 |
| Clinical presentation |  |  |  |
| STEMI, n (%) | 141 (62.1) | 48 (64.0) | 0.77 |
| NSTEMI, n (%) | 86 (37.9) | 27 (36.0) | 0.77 |
| Anterior infarction (LAD), n (%) | 143 (63.0) | 55 (73.3) | 0.10 |
| Killip class ≥ II, n (%) | 10 (4.4) | 14 (18.7) | <0.001 |
| Heart rate (bpm) | 77.5 ± 14.6 | 87.2 ± 17.3 | <0.001 |
| Systolic BP (mmHg) | 132.5 ± 22.8 | 127.1 ± 24.7 | 0.09 |
| GRACE score |  |  |  |
| GRACE score | 136.2 ± 28.5 | 157.8 ± 29.1 | <0.001 |
| Laboratory parameters |  |  |  |
| WBC count (×10⁹/L) | 9.5 ± 3.4 | 11.0 ± 4.3 | 0.004 |
| Absolute neutrophil count (×10⁹/L) | 6.3 ± 3.1 | 8.0 ± 3.9 | <0.001 |
| Absolute lymphocyte count (×10⁹/L) | 2.4 ± 1.4 | 2.0 ± 1.1 | 0.03 |
| NLR | 3.7 ± 3.2 | 5.3 ± 4.2 | 0.001 |
| hs-CRP (mg/L) | 4.2 ± 10.8 | 10.3 ± 28.6 | 0.004 |
| Albumin (g/L) | 43.1 ± 5.8 | 40.1 ± 6.6 | <0.001 |
| CAR | 0.12 ± 0.35 | 0.31 ± 0.89 | 0.009 |
| Creatinine (μmol/L) | 98.5 ± 22.1 | 111.2 ± 47.5 | 0.02 |
| eGFR (ml/min/1.73m²) | 74.5 ± 16.8 | 68.0 ± 20.2 | 0.01 |
| Fasting blood glucose (mmol/L) | 6.7 ± 3.1 | 8.0 ± 4.3 | 0.01 |
| hs-cTnI (ng/L) | 2150 ± 5120 | 5570 ± 8810 | <0.001 |
| SIBS distribution |  |  |  |
| SIBS = 0, n (%) | 145 (63.9) | 26 (34.7) | <0.001 |
| SIBS = 1, n (%) | 69 (30.4) | 32 (42.7) | 0.05 |
| SIBS = 2, n (%) | 13 (5.7) | 17 (22.7) | <0.001 |
| SIBS ≥ 1, n (%) | 82 (36.1) | 49 (65.3) | <0.001 |
| Echocardiography |  |  |  |
| LVEF (%) | 60.5 ± 8.5 | 56.3 ± 9.9 | <0.001 |
| Coronary angiography |  |  |  |
| Multi-vessel disease, n (%) | 55 (24.2) | 32 (42.7) | 0.002 |
| Treatments |  |  |  |
| Primary PCI, n (%) | 198 (87.2) | 62 (82.7) | 0.33 |
| Thrombolysis, n (%) | 8 (3.5) | 3 (4.0) | 0.85 |
| Conservative treatment, n (%) | 21 (9.3) | 10 (13.3) | 0.32 |
| In-hospital outcomes |  |  |  |
| Length of stay (days) | 7.6 ± 3.3 | 8.7 ± 5.8 | 0.04 |

## Supplementary Table S5. Forward Stepwise Logistic Regression for Elevated NT-proBNP (>75th percentile)

| Variable | β Coefficient | Odds Ratio | 95% CI | *P*-value | Selection Status |
| --- | --- | --- | --- | --- | --- |
| Final model variables |  |  |  |  |  |
| SIBS ≥ 1 (vs. SIBS = 0) | 0.87 | 2.39 | 1.32–4.32 | 0.004 | Entered |
| GRACE score (per 1 point increase) | 0.031 | 1.031 | 1.018–1.045 | <0.001 | Entered |
| hs-CRP (per 1 mg/L increase) | 0.11 | 1.11 | 1.04–1.18 | 0.001 | Entered |
| Candidate variables not retained |  |  |  |  |  |
| Age (per 1 year increase) | — | — | — | — | Not entered |
| Sex (male vs. female) | — | — | — | — | Not entered |
| Albumin (per 1 g/L increase) | — | — | — | — | Not entered |
| CAR (per 0.1 unit increase) | — | — | — | — | Not entered |

Notes: Final equation: Logit(*P*) = –4.12 + 0.87 × (SIBS ≥ 1) + 0.031 × (GRACE score) + 0.11 × (hs-CRP in mg/L); Model performance: apparent AUC = 0.815 (95% CI: 0.737–0.888); Optimism-corrected AUC = 0.798; Entry/stay criteria: entry *P* < 0.10, stay *P* < 0.05. Variance inflation factor (VIF) for all final model variables was < 1.8, indicating no significant collinearity.

## Supplementary Table S6. Performance metrics of the composite logistic regression model at optimal cutoff

| Performance metric | Value | 95% Confidence Interval |
| --- | --- | --- |
| Area under the ROC curve (AUC) | 0.815 | 0.737–0.888 |
| Optimism-corrected AUC | 0.798 | 0.724–0.864 |
| Sensitivity | 0.78 | 0.72–0.84 |
| Specificity | 0.72 | 0.66–0.78 |
| Positive predictive value (PPV) | 0.45 | 0.39–0.51 |
| Negative predictive value (NPV) | 0.92 | 0.88–0.96 |
| Positive likelihood ratio (LR+) | 2.79 | 2.35–3.31 |
| Negative likelihood ratio (LR–) | 0.31 | 0.25–0.38 |

**Notes:** Calibration Metrics: Brier Score: 0.168; Calibration Slope: 0.94 (95% CI: 0.82–1.06); Calibration Intercept: –0.12 (95% CI: –0.45 to 0.21); Hosmer-Lemeshow Goodness-of-Fit: *P* = 0.32. Optimal cutoff was determined by the Youden index. Sensitivity and specificity are reported at the optimal probability threshold for identifying elevated NT-proBNP (>75th percentile, 647 pg/mL).

Supplementary Figure S1. Cross‑validation and bootstrap optimism correction results for the composite logistic regression model.

Supplementary Figure S2. Calibration plot of the composite logistic regression model
